# Simulation of the Periodontal Ligament in Dental Materials Research: A CAD/CAM-Based Method for PDL Modeling

**DOI:** 10.3390/jfb16120429

**Published:** 2025-11-24

**Authors:** Przemysław Kosewski, Juliusz Kosewski, Agnieszka Mielczarek

**Affiliations:** 1 Independent Researcher, 01650 Warsaw, Poland; 2 Department of Conservative Dentistry, Medical University of Warsaw, 02091 Warsaw, Poland

**Keywords:** computer-aided design, experimental model, fixed partial denture, in vitro testing, mechanical testing, periodontal ligament

## Abstract

The periodontal ligament (PDL) is essential for the physiological mobility and load distribution of natural teeth, yet its simulation in mechanical testing remains inconsistent and insufficiently standardized. The absence of a resilient suspension system can alter force transmission, affect failure patterns, and reduce the clinical relevance of in vitro outcomes. This study aimed to develop a reproducible CAD/CAM-based model for PDL simulation that provides elastic suspension of a tooth replica under laboratory conditions. A digitally defined offset was applied around a tooth replica to create a controlled PDL space, which was filled with polyether. To ensure precise seating of the specimens, a 3D-printed positioning device was used. Functional calibration was performed using Periotest measurements to identify the offset that reproduced physiological tooth mobility. A digital offset of 0.85 mm produced a radiographically confirmed polyether layer of 0.86 ± 0.05 mm and yielded Periotest values comparable to natural teeth in the horizontal direction (mean PTV = 2.99 ± 0.92). Vertical measurements demonstrated higher damping (mean PTV = −4.02 ± 0.56), consistent with the anisotropic behavior of natural PDL. The model showed high fabrication accuracy and predictable mechanical behavior, providing a physiologically relevant method for incorporating PDL simulation into laboratory mechanical testing.

## 1. Introduction

Natural teeth are anchored in the alveolar bone via the periodontal ligament (PDL)—a thin layer of connective tissue approximately 0.1–0.3 mm thick that fills the space between the tooth root and the alveolar bone [1]. It is composed of collagen fibers embedded in a ground substance and contains interstitial fluid, which imparts its viscoelastic properties [2]. This means that the PDL undergoes slight deformation under applied forces, but its stiffness increases with the magnitude and rate of loading. Under typical masticatory forces of approximately 70 N, the PDL allows for root deflection in the range of 0.03–0.15 mm [1]. Under increasing loads, the PDL exhibits strain-dependent stiffening, as collagen fibers progressively stretch, leading to enhanced stress transfer to the surrounding alveolar bone. [3] This damping mechanism protects the tooth and its surrounding structures from trauma, as the PDL absorbs energy and prevents stress concentration within the root itself [4].

Modern dental treatment concepts are increasingly based on biomimetic principles, aiming to restore the function of the stomatognathic system in a manner that closely replicates natural conditions. A fundamental principle in the rehabilitation of the stomatognathic system is the use of restorative materials that mimic the behavior of natural tissues during oral functions—such as chewing, occlusion, parafunctions, and voice articulation—and that can adapt over time to changing occlusal conditions, similarly to enamel, dentin, or bone tissue [5,6,7]. An additional challenge arises in replicating the function of natural teeth with dental implants used to replace missing teeth. Unlike natural teeth, implants osseointegrate without a PDL, resulting in markedly different biomechanical properties. Lacking the PDL, the implant is rigidly anchored and virtually immobile. Studies have shown that a loaded implant generates higher stress levels in the surrounding bone compared to a natural tooth suspended by the periodontal ligament apparatus [2]. Therefore, the design of biomimetic implant-supported restorations aims to compensate for the reduced ability to dissipate energy in comparison to natural teeth [2].

Some in vitro studies of stomatognathic function have shown that the laboratory model is often simplified by embedding tested teeth or prosthetic materials in a rigid medium (e.g., acrylic resin or plaster) without simulating the PDL. Such models behave similarly to ankylosed teeth, exhibiting no physiological mobility [8]. Omitting the role of the PDL in such studies may lead to distorted mechanical test results.

Previous in vitro investigations have shown that the influence of PDL simulation varies considerably depending on the type of mechanical test performed—being most pronounced in fixed partial dentures (FPDs) [9,10,11], post-and-core systems [12,13] and wear [14,15], while generally less significant in single-crown fracture resistance tests [16,17].

In light of these discrepancies, there is a growing trend in laboratory research to incorporate PDL simulation [18]. The aim is to replicate, within experimental models, the tooth’s ability to undergo slight displacement under load, as observed under in vivo conditions [18]. Accurate replication of these characteristics should result in a stress distribution similar to that observed in nature, thereby enhancing the clinical relevance and translatability of in vitro findings.

Various techniques have been described in the literature to mimic the function of the PDL. However, a standardized protocol for fabricating experimental models that replicate the PDL is still lacking, and the existing approaches show considerable heterogeneity.

In view of these limitations, there is a need for experimental models that reproduce the elastic behavior of the PDL in a standardized and reproducible manner, while remaining compatible with common mechanical testing workflows.

Therefore, the primary objective of this study was to develop and functionally validate a CAD/CAM-based experimental model for PDL simulation around a single-rooted tooth replica, using a polyether layer with digitally controlled thickness calibrated to physiological tooth mobility. A focused overview of current PDL simulation approaches, summarized in Appendix A, was used to contextualize key design choices and to derive practical recommendations for implementing PDL analogs in future in vitro studies.

## 2. Materials and Methods

### 2.1. CAD/CAM-Based Method for the PDL Simulation

A tooth model was digitally designed in SolidWorks 2016 SP 5.0 (Dassault Systèmes SolidWorks Corporation, Vélizy-Villacoublay, France) based on the average dimensions of a maxillary second premolar [19]. The abutment was digitally designed to replicate a tooth prepared for a full lithium disilicate crown, following manufacturer guidelines with a 2 mm occlusal reduction and 1.5 mm axial reduction at the level of crown margin. The convergence angle was set to 8°. The tooth replica was milled from a hybrid ceramic material with mechanical properties comparable to natural dentin [20,21,22,23] (Ambarino High Class, Creamed GmbH & Co., Marburg, Germany). Comparative mechanical data for Ambarino High Class and human dentin are provided in Table 1.

Cubic PMMA (polymethyl methacrylate) (Aidite Clear PMMA, Aidite Technology Co., Ltd., Qinhuangdao, China) blocks measuring 20 × 12 × 12 mm were milled with a cavity left for an artificial socket, generated by digitally enlarging the tooth model and subtracting it from the PMMA block. PMMA was selected because its elastic modulus (mean 2.88 ± 0.01 GPa) [24] is comparable to that of mandibular bone (approximately 3 GPa) [25], ensuring mechanical behavior similar to the supporting structures in vivo.

All components were fabricated using a 5-axis milling unit (RS-Team RS5, RS-Team S.C., Otwock, Poland). Hybrid ceramic specimens were milled under wet conditions with a 1 mm diamond bur at a spindle speed of 60,000 rpm, feed rate of 2.0 mm/s, and a layer thickness of 0.02 mm. PMMA blocks were milled under dry conditions using a carbide bur, with a spindle speed of 40,000 rpm, a feed rate of 3.0 mm/s, and a layer thickness of 0.05 mm.

The positioning devices were fabricated with a stereolithography printer (Formlabs 3, Formlabs Inc., Somerville, MA, USA) using NextDent Model 2.0 resin (Vertex-Dental B.V., Soesterberg, The Netherlands) at 50 µm layer thickness, followed by washing in isopropyl alcohol for 10 min and UV post-curing for 30 min at 60 °C.

The CAD design files for the tooth, socket, and positioning device are openly available on Zenodo (Dataset, DOI: 10.5281/zenodo.17392949) to ensure reproducibility of the CAD/CAM workflow.

According to established methodology, a polyether material (Impregum Penta, 3M ESPE, St. Paul, MN, USA) was used to replicate the viscoelastic properties of the PDL [11,26].

The appropriate space for the polyether layer was determined by fabricating a series of blocks with increasing socket dimensions to achieve vibration-damping properties comparable to natural teeth. Five blocks were fabricated for each of the following ligament space dimensions: 0.25 mm, 0.5 mm, 0.75 mm, and 0.85 mm. The damping capacity of tooth replicas seated in their sockets was evaluated using a Periotest Classic device (Medizintechnik Gulden, Modautal, Germany).

Periotest values (PTVs) range from −8 to +50, where lower or negative readings indicate higher damping capacity and greater stability (as in osseointegrated implants or ankylosed teeth), whereas higher positive readings correspond to reduced damping and increased tooth mobility [27].

**Figure 1:**
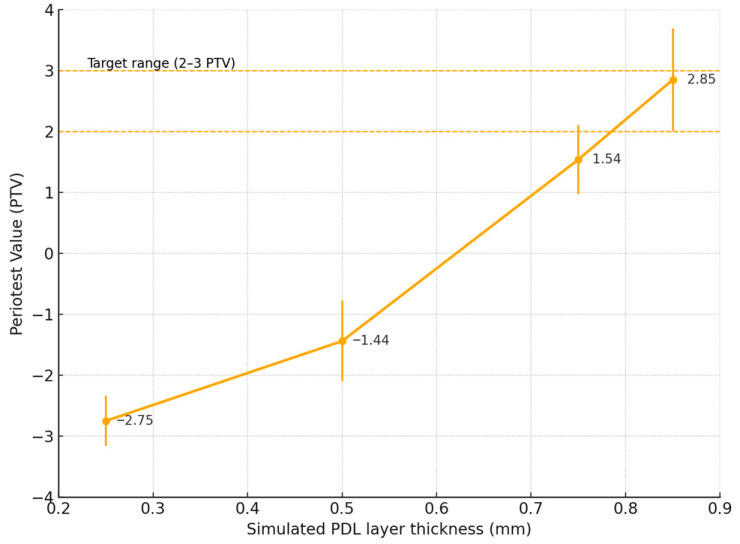
Calibration curve showing the relationship between simulated PDL layer thickness and Periotest values, with error bars representing standard deviations (SD) and the dashed lines indicating the target physiological range of 2–3 PTV.

The objective was to obtain PTVs within the physiological range of 2–3, corresponding to the mobility of natural maxillary teeth (mean value approximately 2.5 PTV) [27]. A PDL space of 0.85 mm provided vibration damping within this range, confirming its suitability for the experimental model. The relationship between simulated PDL thickness and PTV is presented in Figure 1.

To position the tooth roots within the blocks, 3D-printed resin positioning devices were used (NextDent Model 2.0, Vertex-Dental B.V., Soesterberg, The Netherlands). The positioning cube included the following:

- A central socket for the coronal part of the replica, ensuring fixed orientation and maintaining the root at a specific distance from the socket base;
- A 2 mm vertical clearance around the socket for excess polyether material during placement;
- An outer flange matching the block’s contour with a 0.05 mm offset, providing alignment and ensuring the root axis was parallel to the socket’s long axis. The 10 mm-high flange acted as a guide for reproducible root placement within the polyether mass.

The internal surfaces of the socket and the root were coated with polyether adhesive (Polyether Adhesive, 3M ESPE, St. Paul, MN, USA) according to the manufacturer’s instructions. The polyether material was injected into the socket with slight excess. During injection, the dispenser tip was positioned at the bottom of the socket and kept immersed in the material while filling, to prevent air entrapment and ensure uniform layer formation. The tooth replica was fixed in the positioning device, which was then pressed onto the PMMA block until full contact was achieved. Setting of the elastic material was carried out under standard room conditions (approximately 22–24 °C, without controlled humidity). Afterwards, the positioning device was removed, and excess polyether was trimmed flush with the PMMA surface using a scalpel blade.

The prepared models were scanned. Lithium disilicate crowns (IPS e.max CAD, Ivoclar Vivadent, Schaan, Liechtenstein) were designed digitally, milled, and crystallized according to the manufacturer’s instructions.

**Figure 2:**
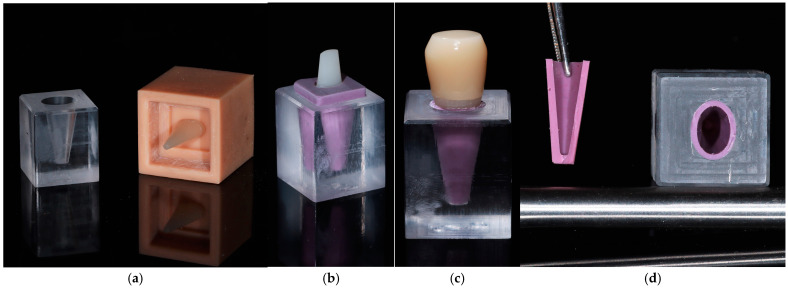
(**a**) PMMA block with a milled socket and a tooth replica positioned using a 3D-printed positioning device; (**b**) Tooth replica inserted into the socket with a polyether layer simulating the PDL; (**c**) Tooth replica with a lithium disilicate crown cemented in place; (**d**) Cross-sectional view of the PDL-mimicking layer.

Before cementation, the intaglio surfaces of the lithium disilicate crowns were etched with 4.5% hydrofluoric acid for 20 s, rinsed with water, and treated with a universal adhesive (Single Bond Universal, 3M ESPE, St. Paul, MN, USA). The hybrid ceramic abutments were air-abraded with 50 µm aluminum oxide (Al_2_O_3_) particles at 2 bar pressure from a distance of approximately 10 mm for 10 s, then thoroughly rinsed with water and air-dried before applying the same adhesive system. A dual-cure resin cement (RelyX Ultimate, 3M ESPE, St. Paul, MN, USA) was applied to the internal surface of each crown, which was then seated under constant finger pressure. Excess cement was carefully removed, followed by light curing for 20 s from each surface, and final polymerization was allowed to occur chemically. Care was taken to prevent air entrapment during cement application to minimize potential damping variability associated with interfacial voids. The experimental model is presented in Figure 2.

A total of 15 specimens were fabricated using the described CAD/CAM-based method. The specimens originated from the same experimental set used in our previous investigation on the effect of abutment rigidity on the wear resistance of lithium disilicate ceramics [15]. The number of specimens per group was originally determined through a power analysis (Statistica 13.3, StatSoft GmbH, Hamburg, Germany), using an independent samples t-test, assuming a 20% intergroup difference based on previously published data, with α = 0.05 and a statistical power of 0.8.

The completed models were evaluated using the Periotest device. Measurements were performed horizontally from the buccal, palatal, mesial, and distal directions, and vertically from the occlusal surface, with three repetitions per direction. The average value was calculated for each plane.

**Figure 3:**
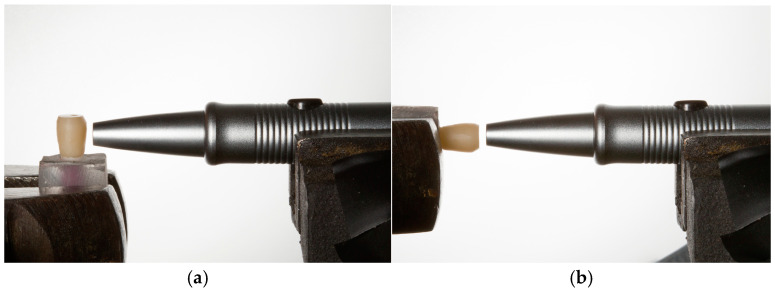
Periotest measurement setup. (**a**) Horizontal orientation: the tapping rod positioned perpendicular to the long axis of the tooth replica. (**b**) Vertical orientation: the specimen rotated by 90°, with the tapping rod perpendicular to the occlusal surface.

During Periotest evaluation both the device and the tested specimens were secured in a metal vise to ensure reproducible alignment and eliminate operator-dependent variability. For horizontal measurements, the tapping rod was oriented perpendicular to the long axis of the tooth replica (Figure 3a), whereas for vertical measurements, the specimen was rotated by 90°, and the handpiece was positioned perpendicular to the occlusal surface (Figure 3b). The tip of the Periotest rod was maintained at a constant distance of 1.5 mm from the tested surface. The device was functionally tested prior to use according to the manufacturer’s recommendations. Each measurement consisted of 16 impacts performed over 4 s, following the standard device settings. All measurements were conducted by the same trained operator to avoid inter-operator variability.

**Figure 4:**
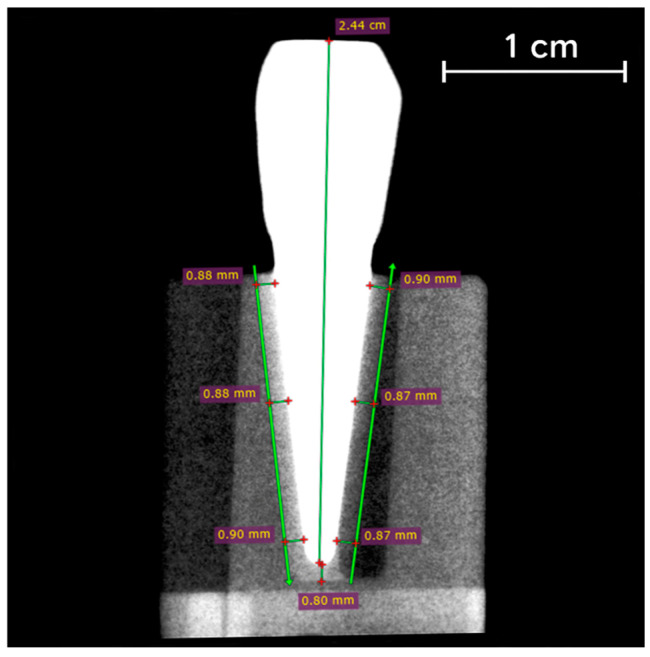
X-ray measurements of the PDL-simulating layer in the CAD/CAM-based experimental model.

Radiographic evaluation was performed using the paralleling technique, with radiographs obtained using a RXDC Hypersphere (Cefla s.c., Imola, Italy) and Rinn XCP-DS FIT positioners (Dentsply Sirona, Charlotte, NC, USA). Exposure parameters were set to 60 kV and 7 mA, with an exposure time of 0.16 s and a source-to-object distance of 10 cm. Images were obtained in both the sagittal and coronal planes. The periodontal space was measured at three points along the root and at the apex (Figure 4) using RadiAnt DICOM Viewer 2025.1 (Medixant, Poznań, Poland). All measurements were calibrated based on the actual specimen dimensions.

### 2.2. Statistical Analysis

Statistical analysis was performed using IBM SPSS Statistics version 29.0 (IBM Corp., Armonk, NY, USA). The Shapiro–Wilk test was used to assess the normality of data distribution. Because the data were not normally distributed, the Kruskal–Wallis test was used to evaluate intergroup differences. Statistical significance was set at α = 0.05. Effect size (ε^2^) and 95% confidence intervals were calculated. Results are reported as descriptive and inferential statistics.

## 3. Results

### Experimental Model Measurements

**Figure 5:**
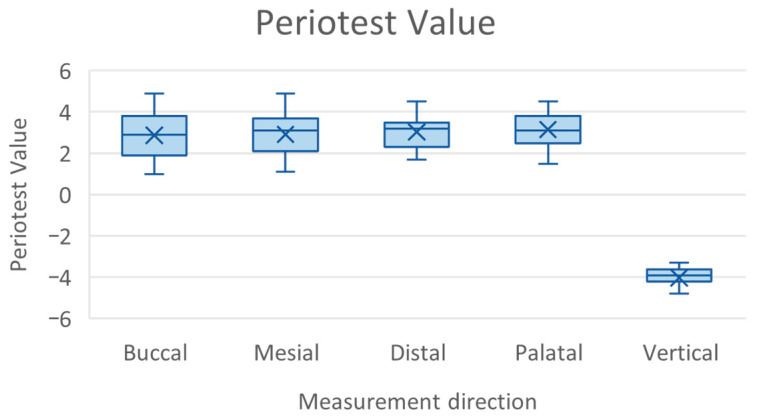
Mean Periotest values in each direction.

The mean PTV in the horizontal plane was 2.99 (SD = 0.92), while in the vertical direction it was −4.02 (SD = 0.56). Average values for each measurement direction are presented in Table 2 and Figure 5 The Kruskal–Wallis test revealed no statistically significant differences between horizontal directions (*p* = 0.460, H(3) = 2.588, ε^2^ = 0.00). When the vertical direction was included, a significant overall difference was detected among all orientations (H(4) = 109.272, *p* < 0.001, ε^2^ = 0.48). Dunn–Bonferroni post hoc analysis confirmed that vertical PTV values were significantly lower than those in each horizontal direction (*p* < 0.001). The results are presented in Table 2.

**Figure 6:**
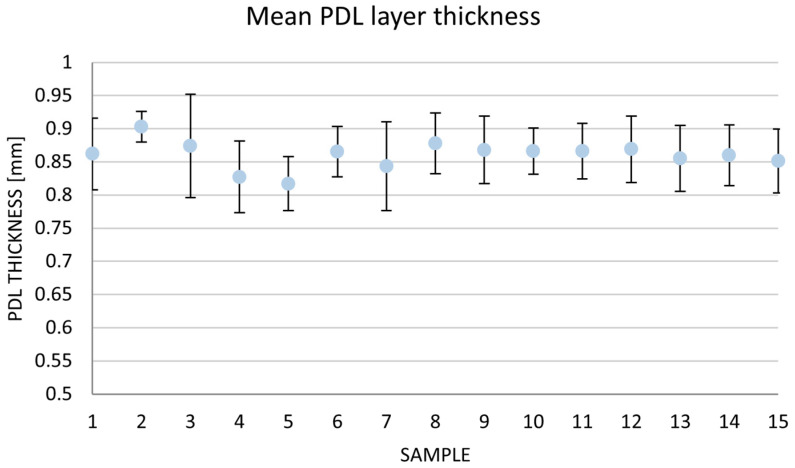
Mean PDL layer thickness.

Measurements of polyether layer thickness simulating the PDL around the root in each model are shown in Table 3 and Figure 6. The mean layer thickness was 0.86 mm (SD = 0.052 mm).

## 4. Discussion

### 4.1. The Influence of PDL Simulation on Mechanical Testing Outcomes

A focused review of the literature was performed to contextualize currently used methods of periodontal ligament simulation. The search strategy, selection criteria, and full summary of findings are presented in Appendix A. Previous studies have shown substantial heterogeneity in the methods used to simulate the PDL. This variation concerned not only the diversity of fabrication techniques and materials employed, but also significant inconsistencies in the documentation of these methods. Many studies lacked a detailed description of model preparation protocols [12,13,18,28,29,30], limiting reproducibility and complicating cross-study comparisons. The inclusion of our previous study [15] carries an inherent risk of bias; therefore, its findings were interpreted with caution to maintain objectivity in the comparative synthesis.

Only a limited number of studies in the available literature included direct comparisons between models with and without PDL simulation, showing that its inclusion significantly influenced the outcomes of selected types of in vitro tests—particularly those assessing fixed partial dentures (FPD) [9,10,11], post-and-core restorations [13,26], material wear [14,15], and fatigue performance [10,28]. The presence of an elastic layer affected both force distribution and the predominant failure mechanisms. Studies including control groups without simulated PDL demonstrated that the absence of a resilient suspension system resulted in significantly different outcomes.

In fracture resistance testing of single crowns, the influence of simulated PDL appears to be less pronounced, although the number of studies directly comparing models with and without PDL remains limited [16,17].

In contrast, tests evaluating the load-bearing capacity and fracture resistance of fixed partial dentures—both retentive and adhesive types—consistently show that the inclusion of a simulated PDL leads to significantly reduced structural strength. This finding has been reported by multiple authors. Rosentritt et al. demonstrated that simulating the PDL reduced the fracture resistance of all-ceramic FPDs by 40–70%. Moreover, the presence of an elastic foundation caused the fracture patterns to more closely resemble those observed under clinical conditions [10,11]. These results were further corroborated by Waldecker et al., who combined physical experimentation with finite element validation, demonstrating that the absence of an elastic PDL led to an overestimation of adhesive FPD strength by 50–95% [9]. This suggests that testing FPDs on rigid abutments without simulating the PDL yields unrealistically high strength values and produces fracture locations and patterns that deviate from those occurring in vivo. The inclusion of a resilient foundation modifies stress transmission pathways by allowing limited bending and torsional deformation of the supporting structures, thereby producing fracture patterns more consistent with clinical failures [10]. Consequently, drawing conclusions from in vitro tests using rigidly fixed abutments may result in clinical applications where the inherent weakness of the construction only becomes apparent upon failure in the oral environment.

In studies on post-and-core restorations, the presence of simulated PDL resulted in lower measured fracture resistance and different fracture patterns compared to specimens mounted in rigid substrates. Hayashi et al. observed that embedding teeth directly in acrylic resin created an artificial “ferrule effect,” which led to overestimated fracture resistance and fracture patterns not representative of clinical conditions—predominantly affecting the coronal region rather than the root [13]. Soares et al. found that PDL simulation significantly influenced the type of observed fractures (with higher incidence of root fractures), but had less impact on the maximum load recorded [26]. Some studies, however, did not report significant differences in fracture resistance between models with and without PDL simulation [31,32]. Nevertheless, most authors emphasize that stress distribution within the root is altered by the presence of a simulated PDL and, therefore, recommend its inclusion in the mechanical testing of post-and-core systems [12,13,26,31,33,34,35,36].

Evidence related to the wear resistance of prosthetic materials in the presence of simulated PDL is limited. Rosentritt et al. experimentally demonstrated that implant-supported crowns may exhibit greater wear and cause more material loss on opposing teeth than crowns supported by teeth with simulated PDL [14]. The findings from our 2023 study are consistent with these observations [15]. A larger sample size enabled quantitative assessment, confirming that differences in material wear may be significant. The comparison included lithium disilicate crowns on tooth replicas with simulated PDL, crowns on implants, and pure material specimens embedded in acrylic resin. The volumetric material loss was measured at 0.107 mm^3^, 0.166 mm^3^, and 0.322 mm^3^, respectively. These results indicate that prosthetic material wear is influenced by the damping properties of the PDL and likely also by other components of the reconstruction, such as the cement layer. Considering the role of the PDL is important in the design of prosthetic materials intended to biomechanically approximate implant-supported restorations to natural teeth. Accounting for this factor may help develop materials that compensate for the reduced energy dissipation associated with the rigid abutment represented by the implant [15]. Moreover, the inclusion of PDL simulation is essential when testing prosthetic materials on natural teeth, as it enables a more accurate replication of the physiological load distribution and tooth mobility, thereby allowing materials to better mimic the natural wear behavior of enamel.

In fatigue tests (thermocycling and mechanical loading, TCML), the presence of the PDL affected not only the total number of cycles to failure but also the type of damage observed. Aboushelib et al. reported that elastic fixation prevented the occurrence of cone cracks and concentric damage patterns typically associated with overly rigid models—an effect attributed to the realistic replication of clinical conditions [28]. Likewise, Rosentritt et al. demonstrated that PDL simulation introduced additional bending and torsional forces on prosthetic bridge structures, which contributed to the material aging effect observed during thermomechanical loading; these effects were less pronounced in rigid configurations [10]. In contrast, Nawafleh et al. found no statistically significant differences between models with and without PDL simulation for zirconia crowns [17]. Therefore, the aging effect appears to be more pronounced in the presence of an elastic foundation in FPDs, where it accelerates the material degradation. In the case of single crowns, some authors have suggested that PDL simulation may have a protective effect against extreme overload, although its impact on overall strength appears less evident. While many researchers investigating cyclic loading in restorations involving post-and-core systems have implemented PDL simulation, its influence in this context has not been directly compared between models with and without resilient fixation.

Regarding fracture resistance testing of crowns under static load, the number of studies comparing models with and without PDL simulation is limited. In the study by Preis et al., fracture resistance was tested in crowns placed on implants and on teeth with simulated PDL, with no statistically significant differences in material strength observed between groups [16]. Similarly, Nawafleh et al. reported no significant differences in cyclic load resistance or fracture strength when comparing zirconia crowns on models with and without PDL simulation [17]. These findings suggest that, for single crowns, the influence of PDL simulation on fracture resistance testing is minimal.

### 4.2. Methods for Simulating the PDL

The most commonly used method for simulating PDL involves manually creating space around the tooth root by dipping the root into molten wax. This technique was first described in 1998 [37]. After placing the tooth in a block and removing the wax, the resulting space was filled with an elastomeric material such as polyether or silicone. The primary advantage of this method is its simplicity; however, the main limitation is the lack of control over the uniformity of the simulated PDL layer. According to various authors, the thickness of the simulated PDL layer may range from 0.00 mm to 0.42 mm (with a planned thickness of 0.25 mm [38]), or between 0.3 mm and 0.7 mm as reported by other researchers [39]. Additionally, as noted by the authors themselves, this method is time-consuming and may lead to inconsistencies in specimen preparation [10].

Higher accuracy and continuity of the PDL layer have been achieved through the direct application of the elastic material onto the root surface. Al-Zahrani et al., by directly applying a latex separator, obtained a consistent and continuous PDL layer with a controlled thickness of 0.25 mm (SD = 0.02 mm), compared to an SD of 0.14 mm using the wax method [38]. Similar techniques using different materials were employed by other researchers [34,36,40,41,42,43,44,45,46], applying the elastic material directly to the tooth root. However, in most studies using this approach, there was a lack of data regarding the elastic properties of the materials used, and only one author performed functional validation of the models by measuring their mobility [43]. The main advantages of this approach include its precision and reproducibility. Potential limitations, however, involve the uncertain elastic properties of rubber-based insulating materials or technical elastomers intended for prosthetic models, as well as the time-consuming nature of manually applying multiple material layers when the desired PDL thickness exceeds that of a single application.

In recent years, models based on CAD/CAM technology have been developed, enabling precise control over the design of PDL dimensions in accordance with its expected mechanical characteristics [15,47]. Digital design and manufacturing automate the entire process, reduce production time, and ensure high reproducibility of experimental models. The protocol presented in this study allows for the use of previously validated elastomeric materials to replicate the elastic behavior of the PDL. Tooth replicas can be either fully digitally designed or based on scans of natural teeth. The limitation of this approach, however, is that the use of natural teeth would require the design and fabrication of a custom alveolar socket replica and corresponding positioners for each individual tooth, which significantly increases the time and resources needed to produce such an experimental model.

New approaches are also emerging that utilize 3D printing to fabricate the PDL layer, potentially offering a promising alternative to conventional elastomeric materials. However, these methods have not yet been validated in terms of their mechanical properties, applicability in strength or fatigue testing, nor have they been used in actual studies of prosthetic materials [48].

The most widely used technique for fabricating PDL-simulating models involves creating space around the tooth using an intermediate material (such as wax or foil) or by digitally designing the space for the PDL, followed by inserting the tooth replica into a socket filled with elastic material. Besides the method of creating the space itself, the process of placing the root into the socket also affects the precision and reproducibility of the model. In the vast majority of studies, this step was performed manually [1,4,11,14,16,17,26,32,33,35,44,49,50,51,52,53,54,55,56,57,58], which introduces the risk of misaligning the root axis and generating an uneven PDL layer in the final model. Several studies used positioning devices to ensure precise insertion of the tooth into the socket. These included silicone-based guides [38,41,59], gypsum [10], and 3D-printed positioners [15], the latter of which are described in detail in this article. The use of positioners during tooth insertion is critical for achieving a homogeneous elastic material layer around the root, thereby ensuring a reproducible experimental model. The direct application technique—where elastic material is applied directly onto the tooth root surface eliminates this technical issue.

### 4.3. Materials Used for PDL Simulation, PDL Layer Thickness, and Model Validation

The studies analyzed revealed a lack of a standardized protocol for selecting materials used in physical simulation of the PDL, resulting in a wide variety of approaches and limiting the comparability of results across investigations. The most commonly used category of materials consisted of elastomeric impression materials, with polyether (Impregum, 3M ESPE) being the most frequently applied [10,11,12,14,15,16,18,28,32,33,35,50,51,53,55,56,57,58,60]. Other elastomeric impression materials were also widely used, most commonly condensation silicones and polyvinyl siloxanes. Standard-viscosity formulations were employed in the majority of cases, although some studies utilized low-viscosity (light body) variants [4,13,17,30,47,49,52,54,61,62]. While often simplified in experimental models as linear-elastic, elastomeric impression materials actually exhibit complex viscoelastic and partially nonlinear stress–strain behavior, with polyether being among the stiffest formulations [63]. These materials are designed for high elastic recovery (low permanent set) and display limited strain tolerance. However, their mechanical response differs substantially from that of the natural PDL, which exhibits viscoelastic, anisotropic, and strain rate-dependent behavior [64]. Another significant category of materials comprised technical elastomers such as “gum resin” or “latex rubber milk” [34,36,40,41,42,45], as well as prosthetic insulators [38,43,44,46] and denture relining materials [31].

In a study by Soares et al., the effects of different elastomeric materials on tooth fracture resistance were compared. The results indicated that, while the differences between materials were relatively minor, the presence of the PDL-simulating layer itself had a significant impact on fracture resistance. The authors also reported that polyether exhibited mechanical properties most closely resembling the physiological characteristics of the natural PDL, particularly in terms of elasticity and its capacity to dampen dynamic load [26]. Given its favorable biomechanical behavior and extensive characterization in the literature, polyether appears to be the most suitable material for simulating the PDL in in vitro studies.

In contrast, Sterzenbach et al. evaluated model mobility using different materials —technical polyurethane (Anti-Rutschlack, Kaddi-Lack, Dortmund, Germany), polyether (Impregum Penta, 3M ESPE, Seefeld, Germany), and polyvinyl siloxane (Mollosil, DETAX, Ettlingen, Germany)—and reported significantly different outcomes. Under a perpendicular load of 30 N, the measured displacement values were 24 µm, 246 µm, and 210 µm, respectively. Corresponding Periotest values also varied notably: −5 to −3 for polyurethane, −1 to +5 for polyether, and +3 to +15 for polyvinyl siloxane [1]. These results indicate that polyurethane, which is frequently used in studies involving the direct application of a root-coating layer, may overly stiffen the model and fail to adequately replicate the PDL’s elasticity. A further conclusion drawn from these findings is that elastomeric materials can differ significantly in their elastic and damping properties. This underscores the importance of functional validation of PDL-simulating models prior to their use in mechanical testing.

Most authors determined the thickness of the simulated PDL layer based on anatomical dimensions of the PDL, typically ranging from 0.2 to 0.3 mm. While these values correspond to the physiological width of PDL observed in histological studies, they may not accurately replicate its biomechanical behavior when elastomeric materials are used. Several experimental studies evaluating tooth mobility through functional testing have demonstrated that a polyether layer of this thickness is excessively rigid compared to the natural PDL. Rosentritt et al., using a universal testing machine, reported that the optimal thickness of the polyether layer should be approximately 1 mm [10]. In our own study, conducted with the Periotest device, we confirmed this characteristic and established that a polyether layer thickness of 0.85 mm is required for a single-rooted tooth to achieve physiological mobility and damping capacity [15]. Therefore, the selection of PDL layer thickness in experimental models should not be based solely on anatomical dimensions but rather on functional criteria—specifically the mechanical properties of the used material. Different elastomeric materials require different layer thicknesses to achieve comparable elastic characteristics. Consequently, the optimal width of simulated PDL must be defined individually for each experimental model.

The majority of studies did not include validation of the experimental models against the actual biomechanical properties of the PDL. Only a limited number of authors assessed tooth displacement using a universal testing machine [1,4,8,10] or an electronic displacement transducer [43], or evaluated vibration damping capacity using the Periotest device [1,15,49,61,62]. The lack of such measurements prevents objective assessment of the biomechanical fidelity of the models and substantially limits both the comparability of the findings and their clinical relevance.

Many authors adopted a simplified approach to the selection of materials for simulating the PDL, often basing their choice solely on the elastic modulus of the material. However, direct comparison of the elastic modulus of synthetic materials with the estimated value for the PDL constitutes a significant oversimplification, as the PDL is neither a homogeneous nor a linearly elastic structure [64]. As a biological tissue, the PDL exhibits anisotropic, nonlinear, and viscoelastic properties, which cannot be adequately described by a single numerical value [65]. Furthermore, most data on PDL elasticity is derived from indirect methods such as inverse finite element analysis (FEA), rather than from direct mechanical testing. Consequently, the elastic modulus of the PDL should be regarded as a context-dependent parameter, influenced by loading conditions, temperature, and measurement methodology [66].

Therefore, the selection of a PDL-analog material should not rely solely on its elastic modulus but should also incorporate functional validation within the context of the specific experimental setup. This may include assessments of tooth mobility under load (e.g., using a universal testing machine) or assessing damping capacity (e.g., using a Periotest device), in order to more accurately replicate the in vivo biomechanical behavior of the PDL. Ultimately, experimental models can only approximate the complex interactions among bone, the periodontal apparatus, dental hard tissues, and prosthetic components and should not be regarded as exact reproductions of clinical conditions.

### 4.4. Reporting Checklist for Studies Including PDL Simulation

Despite the growing number of in vitro studies incorporating periodontal ligament (PDL) simulation, there is currently no standardized framework for reporting experimental conditions. The absence of unified methodological reporting hinders cross-study comparison, reproducibility, and meta-analytical synthesis.

Based on the methodological considerations discussed above, a set of core parameters is proposed to serve as a minimum reporting checklist for studies involving physical PDL simulation. These parameters encompass essential aspects of model design, material selection, and validation procedures, which collectively determine the biomechanical fidelity of the experimental setup.

Recommended minimum reporting items include the following:

- PDL fabrication method: method of creating the PDL space and applying the analog (e.g., lost-wax technique, spacer foil, direct coating, or CAD/CAM-based digital offset).
- PDL analog: material type, brand, viscosity
- PDL layer thickness: target value, tolerance, and method of verification (e.g., µCT, optical measurement, or radiograph).
- Functional validation: validation method (e.g., Periotest, static deflection, or mobility assessment) and target range representing physiological tooth mobility.
- Positioning and alignment: description of root-axis alignment procedure, use of positioning devices or jigs.
- Tooth and substrate: type of tooth, number of roots, arch, and substrate material (e.g., natural tooth, PMMA, resin, gypsum).
- Socket material: composition, manufacturer, and method of cavity preparation.
- Cementation and restoration: type of luting agent, restorative material, and surface treatment.
- Aging and loading protocol: thermocycling, mechanical load magnitude, and number of cycles.
- Outcome measures: parameters evaluated (e.g., fracture load, load bearing capacity, wear).
- Uncertainty assessment: identification and quantification of potential error sources (e.g., layer thickness variability, positioning deviation, or material property variation); report standard deviation, confidence intervals, or coefficient of variation (CV) for key parameters.

These items are summarized in Supplementary Table S1, which may serve as a practical reference for future research aiming to standardize the reporting and validation of experimental PDL-simulation models.

### 4.5. A Novel CAD/CAM Model for PDL Simulation

The model presented in this study, based on CAD/CAM technology, demonstrated high geometric precision. The mean simulated thickness of the PDL layer was 0.86 mm (SD = 0.052 mm), closely matching the digitally designed target of 0.85 mm.

The experimental model was intentionally simplified, featuring a uniform PDL layer and an oval root cross-section, in contrast to the variable ligament thickness and irregular root morphology of natural teeth. This simplification was introduced to facilitate model fabrication and to enable precise, repeatable manufacturing through a standardized digital offset procedure. Despite these simplifications, the model accurately reproduced the global elastic response of the periodontal ligament relevant to mechanical testing applications. Future adaptations in different scenarios should account for specific tooth morphologies, e.g., multi-rooted teeth or variable bone levels, which may influence local stress transfer and mobility calibration.

The established 0.85 mm polyether layer thickness represented the optimal configuration for this specific material (Impregum Penta) and the geometry of a single-rooted maxillary premolar. However, the relationship between the PDL analog thickness and resulting damping behavior is both material- and geometry-dependent. Variations in tooth type, root morphology, or the elastic modulus of the selected material may require individual adjustment of the simulated PDL layer to achieve physiologic mobility. The target calibration range of 2–3 PTV used in this study corresponds to clinical values for natural maxillary teeth, as reported by Winkler et al. [27], where the overall average Periotest value was approximately 2.5, with a range of 2.1–3.1 PTV. For mandibular teeth, lower values (0.8–1.8 PTV) have been observed due to differences in supporting bone quality. Therefore, when developing analogous models, laboratories should calibrate their systems to achieve PTVs within the physiologic range of 1–3, adjusting the PDL layer thickness according to the material properties and tooth geometry.

Functional validation using the Periotest device, which quantifies damping capacity, produced values consistent with those of healthy periodontium. The mean PTV in the horizontal plane was 2.99, within the target range of 2.0–3.0. No statistically significant differences were found among the various horizontal directions (*p* = 0.460), confirming the uniformity of the elastic layer surrounding the roots. In contrast, measurements in the vertical orientation showed markedly higher damping, with a mean PTV of −4.02, compared to the horizontal average, indicating significant anisotropy in the model’s mechanical response (H(4) = 109.272, *p* < 0.001, ε^2^ = 0.48). This behavior corresponds closely with in vivo findings reported by Berthold et al., who observed a median PTV of 1.1 for maxillary canines in the horizontal plane (range: −2.6 to +5.9) and a median of −2.5 in the vertical plane (range: −5.8 to +10.7) [67].

The high reproducibility of the obtained results can be attributed to the application of CAD/CAM technology, which allows precise digital design and control of all model components. A key factor ensuring consistency of the PDL-simulating layer was the dedicated 3D-printed positioning device, which ensured uniform placement of the simulated layer around the tooth roots. This approach minimized inter-sample variability and allowed for a reduction in the required sample size for laboratory testing [15].

Moreover, the model offers advantages in terms of cost-effectiveness and reduced preparation time compared with conventional analog methods. A current limitation lies in the use of tooth replicas fabricated from materials different from natural dental tissues. Although this workflow can be adapted for use with extracted human or animal teeth, it would require the design and fabrication of customized models and positioning devices for each specimen.

## 5. Conclusions

Simulation of the PDL is a critical factor for in vitro studies assessing the mechanical behavior of prosthetic materials and structures. The presence or absence of appropriate PDL simulation in experimental models can substantially impact study outcomes. Despite increasing interest in this topic, significant methodological variability persists in the literature, limiting the comparability of results across studies.

Based on the development of the proposed CAD/CAM-based model, interpreted within the context of relevant literature, the following conclusions can be drawn:

1. The inclusion of a PDL analog is essential when evaluating mechanical properties such as the fracture resistance of fixed partial dentures, the strength of post-and-core restorations, and the wear resistance of prosthetic materials. Omission of this element may distort test outcomes, resulting in either overestimation or underestimation of the actual clinical performance of the material or the prosthetic structure.
2. A comprehensive description of methodologies and materials used in in vitro studies is critical to ensure reproducibility, enable meaningful comparisons across studies, and support the development of reliable and clinically relevant conclusions.
3. Experimental models incorporating PDL simulation should undergo functional validation in addition to geometric verification. This step is essential to confirm that the model accurately replicates the mechanical behavior of the natural PDL, thereby preventing biased or misleading results.
4. The width of the simulated PDL space in experimental models should not be determined solely based on anatomical dimensions. Accurate reproduction requires calibration through functional testing, considering the mechanical properties of the material used to mimic PDL resilience and the anatomical configuration of the tooth replica.
5. The CAD/CAM-based PDL simulation model proposed by the authors demonstrates high reproducibility, ease of fabrication, and accurate emulation of the elastic properties of the natural PDL. This model offers a viable alternative to conventional techniques and may be integrated into various experimental protocols.

## Figures and Tables

**Table 1:** Comparative mechanical properties of human dentin and Ambarino^®^ High Class hybrid ceramic. Data for Ambarino High Class were obtained from the manufacturer’s technical documentation (Creamed GmbH & Co., 2023). Dentin data represent mean ranges reported in the cited studies.

Property	Human Dentin	Ambarino High Class
Elastic modulus (GPa)	16–25 GPa [21,22,23]	10 GPa
Hardness (GPa)	0.4–0.7 GPa [20,23]	0.71 GPa (≈710 MPa)
Compressive strength (MPa)	190–300 MPa [21,22]	500 MPa

**Table 2:** Mean Periotest values in each direction.

Direction	Mean (PTV)	SD (PTV)	Standard Error (PTV)	95% CI (PTV)	Minimum (PTV)	Maximum (PTV)
Buccal	2.88	1.11	0.17	[2.54, 3.21]	1.00	4.90
Palatal	2.91	1.03	0.15	[2.60, 3.22]	1.10	4.90
Mesial	3.04	0.74	0.11	[2.82, 3.26]	1.70	4.50
Distal	3.14	0.75	0.11	[2.91, 3.37]	1.50	4.50
Overall horizontal	2.99	0.92	0.07	[2.86, 3.13]	1.00	4.90
Vertical	−4.02	0.56	0.08	[−4.19, −3.85]	−5.60	−3.30

**Table 3:** Mean PDL layer thickness.

Specimen	Mean (mm)	SD (mm)	Variance (mm^2^)	95% CI (mm)	Minimum (mm)	Maximum (mm)
1	0.86	0.05	0.003	[0.83, 0.89]	0.73	0.92
2	0.90	0.02	0.001	[0.89, 0.92]	0.86	0.95
3	0.87	0.08	0.006	[0.83, 0.92]	0.72	0.99
4	0.83	0.05	0.003	[0.80, 0.86]	0.77	0.94
5	0.82	0.04	0.002	[0.79, 0.84]	0.73	0.86
6	0.87	0.04	0.001	[0.84, 0.89]	0.81	0.92
7	0.84	0.07	0.005	[0.80, 0.88]	0.73	0.95
8	0.88	0.05	0.002	[0.85, 0.90]	0.81	0.94
9	0.87	0.05	0.003	[0.84, 0.90]	0.79	0.95
10	0.87	0.04	0.001	[0.85, 0.89]	0.81	0.92
11	0.87	0.04	0.002	[0.84, 0.89]	0.80	0.92
12	0.87	0.05	0.003	[0.84, 0.90]	0.80	0.93
13	0.86	0.05	0.002	[0.83, 0.88]	0.77	0.93
14	0.86	0.05	0.002	[0.83, 0.89]	0.80	0.94
15	0.85	0.05	0.002	[0.82, 0.88]	0.77	0.93
Overall	0.86	0.05	0.003	[0.85, 0.87]	0.72	0.99

**Figure A1:**
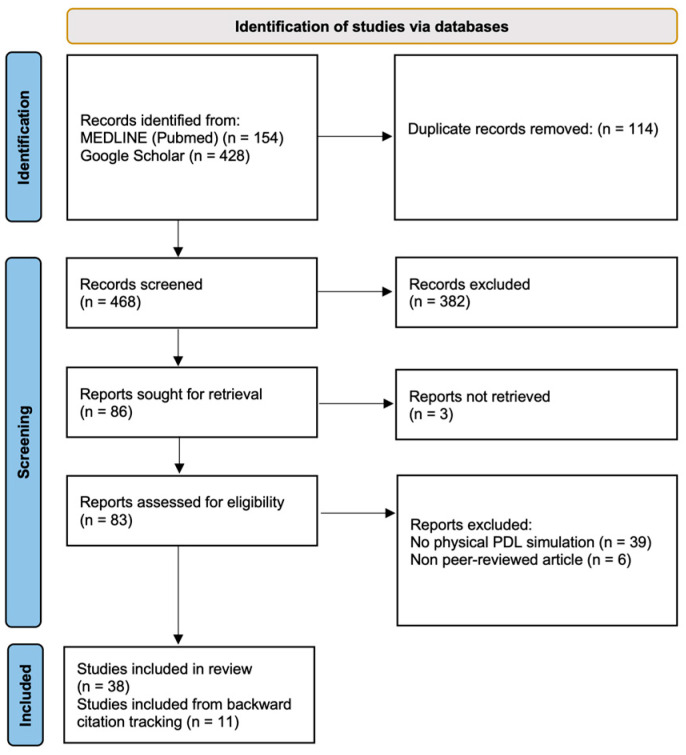
PRISMA 2020 flow diagram of the article selection process.

## Data Availability

The datasets generated and analyzed during the current study, including the STL design files of the tooth, socket, and positioning device, as well as the raw Periotest measurement data used for calibration and functional validation, are openly available on Zenodo at https://doi.org/10.5281/zenodo.17392949 (accessed on 20 September 2025).

## Supplementary Materials

The following supporting information can be downloaded at https://www.mdpi.com/article/10.3390/jfb16120429/s1, Table S1. Minimum Reporting Checklist for Studies Including PDL Simulation; Table S2. Data Extraction Template for PDL Simulation Studies; Table S3. Studies Directly Assessing the Influence of PDL Simulation on Mechanical Test Outcomes.

## Abbreviations

The following abbreviations are used in this manuscript:

CAD/CAM	Computer-Aided Design/Computer-Aided Manufacturing
PDL	Periodontal Ligament
PMMA	Polymethyl methacrylate
PTV	Periotest Value
FPD	Fixed Partial Denture
FEA	Finite Element Analysis
TCML	Thermocycling and Mechanical Loading

## Appendix A

### Appendix A.1. Literature Review Methodology

#### Appendix A.1.1. Search Strategy

A comprehensive literature search was conducted in April 2025 using MEDLINE via PubMed and Google Scholar. Google Scholar was searched to capture grey literature and studies not indexed in MEDLINE. Only studies published in English were considered, which may introduce a potential language bias. No review protocol was pre-registered in PROSPERO or OSF, and, consistent with the scoping review design, no formal quality assessment tool was applied, in line with its exploratory purpose. This review was conducted in accordance with the PRISMA Extension for Scoping Reviews (PRISMA-ScR) guidelines [68]. The objective was to identify studies focusing on the physical simulation of the PDL within in vitro research settings. Two independent reviewers (P.K. and J.K.) performed the search using the following Boolean string:

(periodontal ligament simulation OR PDL simulation OR artificial periodontal ligament OR artificial PDL OR artificial periodontium OR periodontium simulation) AND (prosthetic materials OR resin OR composite OR porcelain OR ceramic OR zirconia OR lithium disilicate OR enamel OR dentin) AND (chewing simulation OR chewing simulator OR fatigue OR wear OR mechanical testing OR mechanical properties OR fracture resistance OR fatigue strength).

#### Appendix A.1.2. Eligibility Criteria

Studies were included if they met the following criteria:

- Published between 2005 and 2025
- Written in English
- Peer-reviewed original research
- Use of physical models simulating the PDL

The 20-year publication window (2005–2025) was chosen to provide a balanced overview of contemporary research while excluding older studies no longer representative of current laboratory methods and materials.

Studies based exclusively on computational simulations (e.g., finite element analysis) or those that did not employ any form of PDL simulation were excluded.

#### Appendix A.1.3. Selection Process

Initial screening was conducted based on titles and abstracts. Articles meeting the inclusion criteria were subjected to full-text review. Additionally, backward citation tracking was performed to identify further relevant studies.

Study selection was carried out independently by two reviewers (P.K. and J.K.). Any disagreements regarding article inclusion were resolved through discussion with a third reviewer (A.M.), who assessed the full text prior to making the final decision. The study selection process is presented in Figure A1.

#### Appendix A.1.4. Data Extraction

The following parameters were extracted from each included study:

- Bibliographic details (authors, year of publication)
- Thickness of the simulated PDL layer
- Material used for PDL simulation
- Material of the abutment tooth
- Technique used for model fabrication
- Tested mechanical parameters
- Type of intervention tested
- Validation of the PDL model (if applicable)
- Assessment of the impact of PDL simulation on study outcomes
- Correlation of findings with in vivo data (if reported)

For clarity, validation procedures were classified as functional—when biomechanical behavior (mobility, damping, or load response) was experimentally verified—or geometric—when dimensional accuracy of the simulated PDL layer was confirmed using imaging or direct measurement. These distinctions are reflected in Appendix A, Table A1.

A data extraction template summarizing all recorded variables (e.g., PDL material, thickness, validation type, test parameters) is provided as Supplementary Table S2.

### Appendix A.2

**Table A1:** Summary of included studies evaluating PDL simulation in in vitro models.

Study (Authors, Year)	Layer Thickness	PDL Simulation Material	Tooth Material	Technique for Artificial PDL	Experimental Model Validation	Parameters Tested	Treatment Type	Influence of PDL on Results	In Vivo Measurements Correlation
Aboushelib (2013) [28]	0.6 mm	Polyether impression material (Impregum; 3M ESPE, St. Paul, MN, USA)	Composite (Z250, 3M ESPE, St. Paul, MN, USA)	Polyether injection into the resin socket—technique not specified	Not specified	Cyclic loading resistance	Zirconia single crown	Lower peak stress in tested materials (lack of cone cracks typical for overloading in the testing environment)	Comparable survival rate
Alqarni et al. (2024) [48]	0.2 mm	3D printing resin—Flexible resin V2 (Formlabs Inc., Somerville, MA, USA)	Human teeth	PDL constructed using CAD/CAM process: root surface scanned and digitally offset 0.2 mm; PDL 3D-printed in flexible resin and fitted to the root, alveolar socket not mentioned	Not specified	Feasibility of digital workflow to fabricate PDL	None	Not specified	Not specified
AlZahrani & Richards (2018) [38]	0.25 mm	Latex rubber die-spacer (Rubber-Sep, Kerr Dental, Brea, CA, USA)—direct technique; light-body silicone impression material (Imprint 4, 3M ESPE, St. Paul, MN, USA)—wax technique	Human teeth	20 coats of paint-on latex rubber applied directly to the root (12 µm each) using a brush, tooth attached to a vertical rod with sticky wax to maintain position, embedded in acrylic resin (ProBase Cold, Ivoclar Vivadent, Schaan, Liechtenstein), comparison with the wax technique with teeth inserted with a silicone positioner	Geometric: Micro-CT—layer thickness measurement	Reproducibility and uniformity of PDL layer thickness (micro-CT analysis)	None	Not applicable	Not applicable
Berthold et al. (2011a) [61]	Not specified	Silicone (not specified) or silicone with rubber foam (not specified)	Stainless steel	Custom aluminum jaw model with alveolar sockets, silicone (not specified) or silicon with rubber foam (not specified) for increased mobility, apical screw for mobility adjustment	Functional: Universal testing machine—deflection measurement; Periotest	Tooth mobility: static (universal testing machine) and dynamic (Periotest)	Splint	Not applicable	Comparable damping capacity (Periotest)
Berthold et al. (2011b) [62]	Not specified	Silicone (not specified) or silicone with rubber foam (not specified)	Stainless steel	Custom aluminum jaw model with alveolar sockets, silicone (not specified) or silicon with rubber foam (not specified) for increased mobility, apical screw for mobility adjustment	Functional: Periotest	Different splint designs rigidity	Splint	Not applicable	Not specified
Boeckler et al. (2008) [49]	0.25 mm	Silicone impression material (Flexistone, Detax, Ettlingen, Germany)	Human teeth	Teeth coated with a wax sheet and embedded in an epoxy resin (Technovit 5000, Heraeus Kulzer, Hanau, Germany), wax removed, socket filled with silicone (Flexistone, Detax, Ettlingen, Germany), specimens reinserted manually	Functional: Periotest	Crowns marginal gap	Tooth-implant supported fixed partial denture	Not applicable	Not specified
Bömicke et al. (2017) [59]	Not specified	Heat-shrink tubing (Protect, Bahag AG, Mannheim, Germany), Light body polyvinylsiloxane impression material (Flexitime Correct Flow, Heraeus Kulzer GmbH, Hanau, Germany) in apical area	Cobalt chromium alloy	Metal abutments with roots covered with heat-shrink tubing and apically sealed with polyvinylsiloxane impression material; embedded in acrylic resin (Technovit 4071; Heraeus Kulzer GmbH, Hanau, Germany) in aluminum blocks, teeth inserted using silicone positioners	Not specified	Load-bearing capacity, fracture patterns under dynamic and static loading	Zirconia and metal-ceramic inlay-retained and wing-retained resin bonded fixed partial dentures	Not specified	Similar failure modes as in clinical conditions
Bruschi-Alonso et al. (2010) [50]	0.2–0.3 mm	Polyether impression material (Impregum; 3M ESPE St. Paul, MN, USA)	Bovine teeth	Roots dipped in melted wax, afterwards embedded in acrylic resin (Vipi Flash; Vipi Ind. Com., Pirassunununga, Brazil), wax removed, socket filled with polyether (Impregum), specimens reinserted manually	Not specified	Impact strength (fracture resistance at high speed impact)	Tooth fragment reattachment	Not specified	Not specified
Clausen et al. (2010) [40]	Not specified	Other elastomer/technical—gum resin (Anti-Rutsch-Lack, Wenko-Wenselaar GmbH, Hilden, Germany)	Human teeth	Roots coated directly with gum resin layer and embedded in acrylic resin (Technovit 4000, Heraeus Kulzer, Hanau,, Germany), roots secured in resin by 0.9 mm stainless steel bar in apical third of the root	Not specified	Survival under cyclic loading; fracture resistance	Ceramic overlay	Not specified	Not specified
Clavijo et al. (2009) [51]	0.2–0.3 mm	Polyether impression material (Impregum; 3M ESPE St. Paul, MN, USA)	Bovine teeth	Roots dipped in melted wax, afterwards embedded in polystyrene resin (Cristal, Araquímica, Araçariguama, Brazil), wax removed, socket was filled with polyether (Impregum), specimens inserted manually	Not specified	Root fracture resistance	Intraradicular post	Not specified	Comparable fracture resistance
Dulaimi & Al-Hashimi (2005) [52]	Not specified	Silicone impression material (DoriDent, Dr Hirschberg GmbH, Vienna, Austria)	Human teeth	Roots wrapped in lead foil embedded in acrylic resin (Medicus Cold Cure, DMP Ltd., Attiki, Greece), lead foil removed and silicone material painted on the root, specimens inserted in the socket manually	Not specified	Endodontic spreader load and penetration depth	Endodontic treatment	Not specified	Not specified
Fulde et al. (2025) [41]	0.2 mm	Other elastomer/technical—gum resin (Anti-Rutsch-Lack, Wenko-Wenselaar, Hilden, Germany)	Human teeth	Roots coated directly with 0.2 mm gum resin (dipping once and removing excess in apical portion), inserted into wax, 0.9 mm metal bar placed through the root to prevent rotation, specimens embedded in acrylic resin (Technovit 4000, Kulzer, Hanau, Germany), specimens reinserted using silicone positioner	Not specified	Survival after thermocycling and mechanical loading, fracture resistance under vertical load, failure mode, wear behaviour (chewing simulation)	Zirconia overlay	Not specified	Not specified
González-Lluch et al. (2016) [31]	0.2 mm	Polyethyl methacrylate denture relining material (Visco-gel, Dentsply Detrey GmbH, Konstanz, Germany)	Human teeth	Roots coated with Visco-gel layer using a brush, inserted into acrylic resin blocks (Vertex Self-Curing Liquid, Zeist, The Netherlands)	Not specified	Fracture resistance and failure modes: teeth with vs. without PDL simulation	Fiber post retained restoration, single crown/no crown	No significant difference in fracture load between PDL vs. no-PDL (*p* = 0.185)—median (538 N vs. 395 N)—limited number of specimens	Not specified
Hasna et al. (2022) [29]	Not specified	Red wax (not specified)	Human teeth	Roots coated with red wax (not specified), embedded in acrylic resin blocks (JET, Artigos Odontológicos Clássico Ltda., São Paulo, Brazil)—technique not specified	Not specified	Deflection, fracture resistance after artificial aging	Endodontic access restoration	Not specified	Not specified
Hayashi et al. (2006) [13]	0.2 mm	Polyvinylsiloxane impression material (Duplicone, Shofu, Kyoto, Japan)	Human teeth	Roots coated with polyvinylsiloxane, embedded in acrylic resin (Uni-Fast II, GC, Tokyo, Japan)—technique not specified	Not specified	Fracture resistance	Intraradicular post and crown	Without simulated PDL in the preliminary study, fracture resistance was approx. twice as high, acrylic resin in specimens without PDL acted as a ferrule	Not specified
Ille et al. (2023) [30]	Not specified	Polyvinylsiloxane impression material (Elite HD, Zhermack, Badia Polesine, Italy)	Human teeth	Teeth inserted in acrylic cylinder (not specified), apical part of the root embedded in polyvinylsiloxane low consistency impression material (not specified)—technique not specified	Not specified	Compressive strength	Ceramic/composite overlays	Not specified	Not specified
Jalalian & Mirzaei (2009) [33]	0.2 mm	Polyether impression material (Impregum, 3M ESPE, St. Paul, MN, USA)	Human teeth	Roots wrapped in 0.2 mm aluminum foil, embedded in acrylic resin; foil removed, sockets filled with Impregum, specimens repositioned manually	Not specified	Compressive strength	Intraradicular fiber post	Not specified	Not specified
Kolbeck et al. (2008) [18]	Not specified	Polyether impression material (Impregum, 3M ESPE, Seefeld, Germany)	Human teeth	Roots coated with polyether, embedded in resin blocks (Palapress Vario: Heraeus Kulzer, Hanau, Germany)—technique not specified	Not specified	Fracture resistance after chewing simulation	Tooth-tooth and implant-tooth fixed partial dentures	Not applicable	Not specified
Kosewski et al. (2023) [15]	0.85 mm	Polyether impression material (Impregum Penta; 3M ESPE, St. Paul, MN, USA)	Hybrid ceramic (Ambarino High Class, Creamed GmbH & Co., Marburg, Germany)	Teeth and bone constructed using CAD/CAM process: PMMA cubes with milled alveolar sockets digitally enlarged, teeth milled based on digital project, sockets filled with polyether material, specimens inserted in the socket with 3D-printed positioner	Functional: Periotest	Crown material wear	Single ceramic crown	Lower antagonist wear (0.172 mm vs. 0.220 mm), wear depth (0.098 mm vs. 0.186 mm), volume loss (0.107 mm^3^ vs. 0.322 mm^3^)	Not specified
Krummel et al. (2019) [42]	0.25 mm	Other elastomer/technical—gum resin (Anti-Rutsch-Lack, Wenko-Wenselaar GmbH, Hilden, Germany)	Human teeth	Roots coated directly with 0.25 mm gum resin layer and embedded in acrylic resin (Technovit 4000, Heraeus Kulzer, Hanau, Germany), roots secured in resin by 0.9 mm stainless steel bar in apical third of the root	Not specified	Survival rate after dynamic loading; fracture resistance; failure mode analysis	Ceramic overlay	Not specified	Not specified
Marchionatti et al. (2014) [32]	0.3 mm	Polyether impression material (Impregum Soft Medium Body, 3M ESPE, St. Paul, MN, USA) vs. silicone impression material (Express Medium Body, 3M ESPE, St. Paul, MN, USA)	Bovine teeth	Roots dipped in melted wax, afterwards embedded in acrylic resin (Dencrilay, Dencril, Caieiras, Brazil), wax removed, socket filled with simulation material, specimens reinserted manually	Not specified	Fiber posts bond strength, roots fracture resistance	Intraradicular fiber post	No effect	Not specified
Nawafleh et al. (2020) [17]	0.3 mm	Light body elastomeric impression material (3M ESPE, St. Paul, MN, USA)—not specified	Epoxy resin (Exakto-Form, Bredent, Senden, Germany)	Wax layer added to the roots, embedded in in acrylic resin, wax substituted with elastomeric impression material, specimens reinserted manually	Not specified	Fatigue survival and fracture resistance	Single zirconia crown	No effect	Not specified
Oliveira et al. (2022) [53]	Not specified	Polyether impression material (Impregum; 3M ESPE, St. Paul, MN, USA)	Polystyrene resin (Cristal, Araquímica, Araçariguama, Brazil)	Alveolus in acrylic model adapted manually, polyether injected in the socket, specimens reinserted manually	Not specified	Proximal contact force	Direct restoration	Not applicable	Not specified
Pişkin et al. (2008) [54]	Not specified	Light body silicone impression material (Speedex light body, Coltene/Whaledent, Altstätten, Switzerland)	Human teeth	Tooth wrapped in a single layer of aluminium foil, inserted in polystyrene resin, after aluminium foil removal, silicone material injected in the sockets, specimens reinserted manually	Not specified	Fracture resistance	Endodontic treatment	Not specified	Not specified
Preis et al. (2015) [55]	1 mm	Polyether impression material (Impregum, 3M Espe, Seefeld, Germany)	Human teeth	Roots dipped in melted wax, embedded in resin blocks (Palapress Vario, Kulzer, Hanau, Germany), wax removed, socket filled with polyether (Impregum), specimens reinserted manually	Not specified	Fracture resistance and marginal adaptation after thermocycling and mechanical loading under different cementation types	Monolithic zirconia-reinforced lithium silicate single crowns	Not specified	Not specified
Preis et al. (2018) [16]	1 mm	Polyether impression material (Impregum, 3M, Seefeld, Germany)	Human teeth	Roots dipped in melted wax, embedded in resin blocks (Palapress Vario, Kulzer, Hanau, Germany), wax removed, socket filled with polyether (Impregum), specimens reinserted manually	Not specified	Survival after thermocycling and mechanical loading, fracture resistance, effect of restoration type (tooth/implant), polishing vs. glazing	Lithium aluminosilicate single crowns	No effect	Not specified
Puschmann et al. (2009) [43]	0.2 mm	Latex rubber die-spacer (Erkoskin, Erkodent, Pfalzgrafenweiler, Germany)	Cobalt chromium alloy (Wironit; Bego, Bremen, Germany)	Roots of metal teeth coated directly with a 0.2 mm silicone layer; roots embedded in low-melting–point alloy (MCP 70) using a duplication form	Functional: Mobility checked with an electronical displacement transducer (W1T3; HBM, Darmstadt, Germany)—displacement of 50 µm under 20 N of horizontal load	Load-bearing capacity with and without fatigue loading; fracture mode analysis	Ceramic fixed partial denture	Not specified	Not specified
Rathke et al. (2022) [34]	0.25 mm	Other elastomer/technical—latex rubber milk (Suter Kunststoffe, Fraubrunnen, Switzerland)	Human teeth	Roots coated directly with one layer of latex rubber milk (Suter Kunststoffe, Switzerland) and embedded in acrylic resin (Technovit 4071, Heraeus Kulzer, Hanau, Germany)	Not specified	Root fracture resistance and crack formation after chewing simulation and static loading	Intraradicular glass fiber and metal posts	Not specified	Not specified
Ribeiro et al. (2023) [12]	0.3 mm	Polyether impression material (Impregum F, 3M Oral Care, St. Paul, MN, USA)	Bovine teeth	Roots coated with a 0.3 mm polyether layer and embedded in polystyrene resin (Cristal, Araquímica, Araçariguama, Brazil), technique not specified	Not specified	Survival rate and failure mode under cyclic loading	Intraradicular glass fiber post and direct restoration	Different results from studies without PDL simulation	Comparable differences between survival rates of teeth restored with fiber posts vs. no posts, and different fracture modes than in clinical studies
Rosentritt et al. (2006) [11]	1 mm	Polyether impression material (Impregum, 3M ESPE, Seefeld, Germany)	Liquid crystal polymer (LCP) and human teeth	Roots dipped in melted wax, afterwards embedded in PMMA resin (not specified), wax removed, socket filled with polyether (Impregum), specimens reinserted manually	Not specified	Fracture resistance of all-ceramic fixed partial dentures after thermocycling and mechanical loading	Ceramic fixed partial denture	Fracture resistance reduced by up to 70%; omission of PDL simulation leads to overestimation of fracture resistance	Not specified
Rosentritt et al. (2011) [10]	0.75 mm	Polyether impression matrial (Impregum, 3M ESPE, Seefeld, Germany)	Human teeth	Roots dipped in melted wax, embedded in PMMA (Palapress Vario, Kulzer, Hanau, Germany), wax removed, sockets filled with polyether (Impregum), specimens reinserted using repositioning cast	Functional/Geometric: Universal testing machine—deflection measurement; PDL thickness measured after slicing the specimens	Fracture resistance of three-unit fixed partial dentures after thermocycling and mechanical loading	Ceramic fixed partial denture	Fracture force reduced by 40–50% with PDL; no PDL led to overestimation; PDL presence seemed to be responsible for the ageing effect during TCML	Similar modes of failure as in clinical conditions
Rosentritt et al. (2020) [14]	1 mm	Polyether impression material (Impregum, 3M ESPE, Seefeld, Germany)	Human teeth	Roots dipped in melted wax, embedded in resin blocks (Palapress Vario, Kulzer, Hanau, Germany), wax removed, socket filled with polyether (Impregum), specimens reinserted manually	Not specified	Number and depth of wear traces	Zirconia/ceramic single crown	Crown on implants displayed increased antagonistic wear compared to teeth with PDL	Wear patterns similar to clinical findings
Sarafidou et al. (2012) [44]	Not specified	Latex rubber die-spacer (Erkoskin, Erkodent, Pfalzgrafenweiler, Germany)	Polyurethane resin (PUR, Alpha-Die-Top, Schütz Dental, Rosbach vor der Höhe, Germany)	Roots coated directly with latex layer (Erkoskin); embedded manually in polyurethane base	Not specified	Load-bearing capacity after artificial ageing (thermocycling and mechanical loading); fracture mode analysis	Fixed partial denture	not specified	not specified
Sasse et al. (2015) [45]	Not specified directly, reference to previous studies (0.2 mm)	Other elastomer/technical—gum resin (Anti-Rutsch-Lack, Wenko-Wenselaar GmbH, Hilden, Germany)	Human teeth	Roots coated directly with gum resin layer and embedded in acrylic resin (Technovit 4000, Heraeus Kulzer, Hanau, Germany), roots secured in resin by 0.9 mm stainless steel bar in apical third of the root	Not specified	Survival rate after dynamic loading; fracture resistance; failure mode analysis	Ceramic overlay	Not specified	Not specified
Sivieri-Araujo et al. (2015) [35]	0.25–0.3 mm	Polyether impression material (Impregum Soft, 3M ESPE AG, Seefeld, Germany)	Bovine teeth	Roots dipped in melted wax, embedded in polystyrene resin (not specified), wax removed, socket filled with polyether (Impregum), specimens reinserted manually	Not specified	Fracture resistance	Intraradicular post	Not specified	Not specified
Soares et al. (2005) [26]	0.2–0.3 mm	Polyether impression material (Impregum F, 3M-ESPE, Seefeld, Germany)/Polysulfide (Permlastic, Kerr, Brea, CA, USA)/Polyurethane elastomer (Ultra Flex, Solplas, Santo André, Brazil)	Bovine teeth	Roots dipped in melted wax, embedded in self-cured acrylic resin (Jet Clássico, Artigos Odontológicos Clássico Ltda., São Paulo, Brazil)/polystyrene resin (Cristal, Araquímica, Araçariguama, Brazil), wax removed, sockets filled with simulation material, specimens reinserted manually	Not specified	Fracture resistance and fracture modes	None	Presence of PDL simulation significantly altered fracture modes (more root fractures); absence of PDL caused concentration of fractures at coronal region	Fracture modes with PDL simulation similar to clinical fracture patterns
Soares et al. (2011) [56]	0.2–0.3 mm	Polyether impression material (Impregum F, 3M ESPE, St. Paul, MN, USA)	Human teeth	Roots dipped in melted wax, embedded in wax model of the mandible, wax from roots eliminated, wax mandible duplicated in polystyrene resin (Aerojet, Aerojet Fiberglass, São Paulo, Brazil), socket filled with polyether (Impregum), specimens reinserted manually	Not specified	Bone strain under different conditions of bone loss and periodontal splinting	Splint	Presence of artificial PDL allowed more realistic strain distribution	Strain values comparable to cadaveric bone measurements
Souza et al. (2014) [57]	0.2–0.3 mm	Polyether impression material (Impregum Soft, 3M ESPE, Seefeld, Germany)	Bovine teeth	Roots dipped in melted wax, afterwards embedded in polystyrene resin (Cristal, Araquímica, Araçariguama, Brazil), wax removed, socket filled with polyether (Impregum), specimens reinserted manually	Not specified	Fracture resistance	Endodontic irrigation	Not specified	Not specified
Sterzenbach et al. (2011) [1]	0.3 mm	Polyurethane (Anti-Rutschlack, Kaddi-Lack, Dortmund, Germany); Polyether (Impregum Penta, 3M ESPE, Seefeld, Germany); Polyvinylosiloxane (Mollosil, DETAX, Ettlingen, Germany)	Human teeth	Roots coated with 0.3 mm wax, embedded in acrylic resin (Technovit 4004), wax removed, sockets filled with simulation material; specimens reinserted manually	Functional: Periotest; universal testing machine—deflection measurement	Tooth deflection (mobility) under axial and perpendicular loading; damping capacity—Periotest values	None	Not applicable	Mobility similar to clinical conditions
Sterzenbach et al. (2012) [36]	0.1 mm	Other elastomer/technical—gum resin (Anti-Rutsch-Lack, Wenko-Wenselaar GmbH, Hilden, Germany)	Human teeth	Roots coated directly with 0.1 mm silicone (Anti-Rutsch-Lack) and embedded in autopolymerizing acrylic resin (Technovit 4000, Heraeus Kulzer, Hanau, Germany); long axis angled at 135° probably with an index—technique not specified directly, reference to previous study	Not specified	Maximum load capability after thermocycling and mechanical loading; fracture patterns depending on restorative stage	Post-endodontic restorations: fiber post vs. post and core vs. post, core, ceramic crown	Not specified	Not specified
Tanomaru-Filho et al. (2014) [58]	0.25 mm	Polyether impression material (Impregum Soft, 3M ESPE AG, Seefeld, Germany)	Bovine teeth	Roots dipped in melted wax, embedded in polystyrene resin (Cristal, Araquímica, Araçariguama, Brazil), wax removed, sockets filled with polyether (Impregum); specimens reinserted manually	Not specified	Fracture resistance	Intraradicular post/endodontic filling	not specified	Not specified
Waldecker et al. (2019) [9]	Not specified	Heat-shrink tubing (Protect, Bahag AG, Mannheim, Germany), low-viscosity polyvinylsiloxane impression material (Flexitime Correct Flow; Heraeus Kulzer GmbH, Hanau, Germany) in apical area	Cobalt-chromium alloy	Abutment roots covered with heat-shrink tubing sealed apically with polyvinyl siloxane; embedded in aluminum blocks with acrylic resin (Technovit 4071; Heraeus Kulzer GmbH, Hanau, Germany)	Functional: Model deflection measured under load of 100 N (technique not specified)	Validation of finite elements analysis model; stress distribution and fracture resistance under axial and oblique loading	Zirconia inlay-retained fixed partial denture	Absence of simulated resilience led to overestimation of fracture resistance by 50–95%	Good correlation with clinical behaviour
Weigl et al. (2018) [60]	1 mm	Polyether impression material (Impregum, 3M Oral Care, Seefeld, Germany)	Hybrid ceramic (Ambarino High Class, Creamed GmbH & Co., Marburg, Germany)	Roots dipped in wax, embedded in resin blocks (Palapress Vario, Heraeus-Kulzer, Hanau, Germany), sockets filled with polyether—technique not specified in detail, reference to previous studies	Not specified	Survival under thermocycling and mechanical loading, fracture resistance, fracture mode, influence of crown thickness and cement type	Zirconia single crown	Not specified	Not specified
Wong & Botelho (2014) [8]	Not specified	Orthodontic elastic rings (Z-pak Elastics, Ormco Corp., Brea, CA, USA)	Stainless steel	Two or more elastic orthodontic rings placed apically and circumferentially around the tooth analog, rings placed in grooves in the root surfaces, specimens reinserted in metal base holders	Functional: Universal testing machine—deflection measurement	Fatigue bond strength under cyclic loading	Fixed-fixed/cantilever resin-bonded fixed partial denture	Not applicable	More mobility of the roots than in the clinical situation (550 µm vs. 25–100 µm)
Yeslam et al. (2023) [46]	0.55 mm	Latex rubber die-spacer (Erkoskin, Erich Kopp GmbH, Pfalzgrafenweiler, Germany)	Die resin (Mirapont, Hager & Werken GmbH & Co. KG, Germany)	Roots painted directly with liquid latex (Erkoskin), embedded in die resin (Mirapont, Hager & Werken GmbH & Co. KG, Duisburg, Germany), manual insertion into the resin	Not specified	Fracture resistance	Hybrid ceramic single crown	Not specified	Fracture modes different than those in clinical conditions
Zhang et al. (2016) [69]	0.3 mm	Polyvinylsiloxane impression material (Imprint II, 3M ESPE, St. Paul, MN, USA)	Composite resin (Filtek P60, 3M ESPE, St. Paul, MN, USA)	Roots coated with 0.3 mm polyvinylsiloxane and embedded in PMMA support base—technique not specified	Not specified	Validation of finite elements analysis model; crack initiation and propagation under loading	Zirconia inlay and onlay fixed partial denture	Not specified	Not specified
Zhu et al. (2015) [4]	0.2–0.3 mm	Silicone impression material (Honigum-Mixstar Mono, DMG, Hamburg, Germany)	Human teeth	Roots dipped in melted wax, afterwards embedded in epoxy resin (Phoenix; Blue-star Manufacturing, Wuxi, China), wax removed, socket filled with silicone, specimens reinserted manually	Functional: Universal testing machine—compression testing—elastic modulus evaluation	Cyclic loading resistance	Vertical root fracture bonding	PDL simulation significantly increased fatigue resistance (PDL group: 150, 580 ± 21, 570 cycles vs. non-PDL group: 29, 2 [62] ±9, 473 cycles, *p* < 0.001)	Not specified
Zimmermann et al. (2020) [47]	0.1 mm	Polyvinylsiloxane impression material (PRESIDENT light body, Coltène AG, Altstätten, Switzerland)	Composite resin (BRILLIANT Crios CAD/CAM, Coltène AG, Altstätten, Switzerland)	Teeth and bone constructed using CAD/CAM process: teeth and alveolar sockets milled from composite (Brilliant) and (PEEK BreCAM.bioHPP—bredent medical GmbH; Senden, Germany), elastomeric material injected into the socket, specimen insertion not specified	Not specified	Fracture resistance after thermocycling and mechanical loading	Ceramic/composite fixed partial denture	Not specified	Not specified

### Appendix A.3. Literature Review Results

A total of 582 records were identified by searching the Medline and Google Scholar electronic databases. After removal of the 114 duplicates, 468 records were screened based on the title and abstract, resulting in 86 reports sought for retrieval. Three records could not be retrieved because full-text versions were not accessible through institutional or open-access databases; therefore, 83 studies were included for full-text analysis. Of these, 45 studies were excluded after full-text review for the following reasons: lack of physical PDL simulation (*n* = 39) and non-peer-reviewed articles (*n* = 6). A total of 38 articles fulfilled all methodological requirements and were included in the final analysis. Additionally, citation tracking was performed, resulting in the identification of 11 additional relevant articles through the reference lists of the included studies, leading to a total of 49 studies included in the review.

The following data were extracted from the studies: material used for PDL simulation and its thickness, material used to simulate the tooth structure with details on the creation of the artificial PDL-tooth complex; methods of experimental model validation; parameters tested; and type of treatment examined. Furthermore, the influence of PDL simulation on the results and information about the correlation with in vivo measurements were also included. The results of the literature review are presented in Appendix A.2, in Table A1.

A concise summary of studies that directly evaluated the influence of PDL simulation on mechanical test outcomes is provided in Supplementary Table S3. The table includes only investigations in which the effect of PDL presence or absence was explicitly analyzed, listing the test type, PDL analog material, validation approach, and the direction of the observed effect. Studies that performed direct comparisons between models with and without simulated PDL are clearly highlighted, providing a transparent overview of the most methodologically relevant evidence available.

## References

[1] Sterzenbach G., Kalberlah S., Beuer F., Frankenberger R., Naumann M. (2011). In-vitro simulation of tooth mobility for static and dynamic load tests: A pilot study. Acta Odontol. Scand..

[2] Chang H.-H., Yeh C.-L., Wang Y.-L., Huang Y.-C., Tsai S.-J., Li Y.-T., Yang J.-H., Lin C.-P. (2021). Differences in the biomechanical behaviors of natural teeth and dental implants. Dent. Mater..

[3] Wen X., Pei F., Jin Y., Zhao Z. (2025). Exploring the mechanical and biological interplay in the periodontal ligament. Int. J. Oral Sci..

[4] Zhu Y.N., Yang W.D., Abbott P.V., Martin N., Wei W.J., Li J.J., Chen Z., Wang W.M. (2015). The biomechanical role of periodontal ligament in bonded and replanted vertically fractured teeth under cyclic biting forces. Int. J. Oral Sci..

[5] D’Arcangelo C., Vanini L., Rondoni G., Vadini M., De Angelis F. (2018). Wear evaluation of prosthetic materials opposing themselves. Oper. Dent..

[6] Singer L., Fouda A., Bourauel C. (2023). Biomimetic approaches and materials in restorative and regenerative dentistry: Review article. BMC Oral Health.

[7] Zafar M.S., Amin F., Fareed M.A., Ghabbani H., Riaz S., Khurshid Z., Kumar N. (2020). Biomimetic aspects of restorative dentistry Biomaterials. Biomimetics.

[8] Wong T.L., Botelho M.G. (2014). The fatigue bond strength of fixed-fixed versus cantilever resin-bonded partial fixed dental prostheses. J. Prosthet. Dent..

[9] Waldecker M., Rues S., Rammelsberg P., Bömicke W. (2019). Validation of in-vitro tests of zirconia-ceramic inlay-retained fixed partial dentures: A finite element analysis. Dent. Mater..

[10] Rosentritt M., Behr M., Scharnagl P., Handel G., Kolbeck C. (2011). Influence of resilient support of abutment teeth on fracture resistance of all-ceramic fixed partial dentures: An in vitro study. Int. J. Prosthodont..

[11] Rosentritt M., Behr M., Gebhard R., Handel G. (2006). Influence of stress simulation parameters on the fracture strength of all-ceramic fixed-partial dentures. Dent. Mater..

[12] Ribeiro M.T.H., Oliveira G., Oliveira H.L.Q., Mendoza L.C.L., Melo C., Silva Peres T., Soares C.J. (2023). Survival of severely compromised endodontically treated teeth restored with or without a fiber glass post. J. Appl. Oral Sci..

[13] Hayashi M., Takahashi Y., Imazato S., Ebisu S. (2006). Fracture resistance of pulpless teeth restored with post-cores and crowns. Dent. Mater..

[14] Rosentritt M., Schumann F., Krifka S., Preis V. (2020). Influence of zirconia and lithium disilicate tooth- or implant-supported crowns on wear of antagonistic and adjacent teeth. J. Adv. Prosthodont..

[15] Kosewski P., De Angelis F., Sorrentino E., Mielczarek A., Buonvivere M., D’Arcangelo C. (2023). Effect of the abutment rigidity on the wear resistance of a lithium disilicate glass ceramic: An in vitro study. J. Funct. Biomater..

[16] Preis V., Hahnel S., Behr M., Rosentritt M. (2018). In vitro performance and fracture resistance of novel CAD/CAM ceramic molar crowns loaded on implants and human teeth. J. Adv. Prosthodont..

[17] Nawafleh N., Bibars A.R., Elshiyab S., Janzeer Y. (2020). In vitro simulation of periodontal ligament in fatigue testing of dental crowns. Eur. J. Dent..

[18] Kolbeck C., Behr M., Rosentritt M., Handel G. (2008). Fracture force of tooth-tooth- and implant-tooth-supported all-ceramic fixed partial dentures using titanium vs. customised zirconia implant abutments. Clin. Oral Implant. Res..

[19] Kulkarni V., Duruel O., Ataman-Duruel E.T., Tözüm M.D., Nares S., Tözüm T.F. (2020). In-depth morphological evaluation of tooth anatomic lengths with root canal configurations using cone beam computed tomography in North American population. J. Appl. Oral Sci..

[20] Kucher M., Dannemann M., Modler N., Bernhard M.R., Hannig C., Weber M.T. (2021). Mapping of the micro-mechanical properties of human root dentin by means of microindentation. Materials.

[21] Kinney J., Marshall S., Marshall G. (2003). The mechanical properties of human dentin: A critical review and re-evaluation of the dental literature. Crit. Rev. Oral Biol. Med..

[22] Chun K., Choi H., Lee J. (2014). Comparison of mechanical property and role between enamel and dentin in the human teeth. J. Dent. Biomech..

[23] Andrejovská J., Petruš O., Medveď D., Vojtko M., Riznič M., Kizek P., Dusza J. (2023). Hardness and indentation modulus of human enamel and dentin. Surf. Interface Anal..

[24] Di Carlo S., De Angelis F., Brauner E., Pranno N., Tassi G., Senatore M., Bossù M. (2020). Flexural strength and elastic modulus evaluation of structures made by conventional PMMA and PMMA reinforced with graphene. Eur. Rev. Med. Pharmacol. Sci..

[25] Odin G., Savoldelli C., Bouchard P.O., Tillier Y. (2010). Determination of Young’s modulus of mandibular bone using inverse analysis. Med. Eng. Phys..

[26] Soares C.J., Pizi E.C., Fonseca R.B., Martins L.R. (2005). Influence of root embedment material and periodontal ligament simulation on fracture resistance tests. Braz. Oral Res..

[27] Winkler S., Morris H.F., Spray J.R. (2001). Stability of implants and natural teeth as determined by the Periotest over 60 months of function. J. Oral Implantol..

[28] Aboushelib M.N. (2013). Simulation of cumulative damage associated with long term cyclic loading using a multi-level strain accommodating loading protocol. Dent. Mater..

[29] Hasna A.A., Pinto A.B.A., Coelho M.S., de Andrade G.S., Tribst J.P.M., de Castro Lopes S.L.P., Carvalho C.A.T., Borges A.L.S. (2022). Fracture resistance and biomechanical behavior of different access cavities of maxillary central incisors restored with different composite resins. Clin. Oral Investig..

[30] Ille C., Moacă E.-A., Pop D., Goguță L., Opriș C., Pîrvulescu I.L., Avram L., Faur A., Jivănescu A. (2023). Compressive strength evaluation of thin occlusal veneers from different CAD/CAM materials, before and after acidic saliva exposure. Odontology.

[31] Gonzalez-Lluch C., Rodriguez-Cervantes P.J., Forner L., Barjau A. (2016). Inclusion of the periodontal ligament in studies on the biomechanical behavior of fiber post-retained restorations: An in vitro study and three-dimensional finite element analysis. Proc. Inst. Mech. Eng. Part H J. Eng. Med..

[32] Marchionatti A.M., Wandscher V.F., Broch J., Bergoli C.D., Maier J., Valandro L.F., Kaizer O.B. (2014). Influence of periodontal ligament simulation on bond strength and fracture resistance of roots restored with fiber posts. J. Appl. Oral Sci..

[33] Jalalian E., Mirzaei M. (2009). In vitro evaluation of different diameters of quartz fiber posts on root fracture resistance. J. Iran. Dent. Assoc..

[34] Rathke A., Frehse H., Hrusa B. (2022). Vertical root fracture resistance and crack formation of root canal-treated teeth restored with different post-luting systems. Odontology.

[35] Sivieri-Araujo G., Tanomaru-Filho M., Guerreiro-Tanomaru J.M., Bortoluzzi E.A., Jorge E.G., Reis J.M. (2015). Fracture resistance of simulated immature teeth after different intra-radicular treatments. Braz. Dent. J..

[36] Sterzenbach G., Rosentritt M., Frankenberger R., Paris S., Naumann M. (2012). Loading standardization of postendodontic restorations in vitro: Impact of restorative stage, static loading, and dynamic loading. Oper. Dent..

[37] Scharnagl P., Behr M., Rosentritt M., Leibrock A., Handel G. (1998). Simulation of physiological tooth mobility in in-vitro stress examination of dental restorations in the masticator. J. Dent. Res..

[38] AlZahrani F., Richards L. (2018). Micro-CT evaluation of a novel periodontal ligament simulation technique for dental experimental models. Arch. Orofac. Sci..

[39] Heintze S.D., Monreal D., Reinhardt M., Eser A., Peschke A., Reinshagen J., Rousson V. (2018). Fatigue resistance of all-ceramic fixed partial dentures—Fatigue tests and finite element analysis. Dent. Mater..

[40] Clausen J.O., Abou Tara M., Kern M. (2010). Dynamic fatigue and fracture resistance of non-retentive all-ceramic full-coverage molar restorations. Influence of ceramic material and preparation design. Dent. Mater..

[41] Fulde N., Wille S., Kern M. (2025). Fracture resistance and wear behavior of ultra-thin occlusal veneers made from translucent zirconia ceramics bonded to different tooth substrates. J. Esthet. Restor. Dent..

[42] Krummel A., Garling A., Sasse M., Kern M. (2019). Influence of bonding surface and bonding methods on the fracture resistance and survival rate of full-coverage occlusal veneers made from lithium disilicate ceramic after cyclic loading. Dent. Mater..

[43] Puschmann D., Wolfart S., Ludwig K., Kern M. (2009). Load-bearing capacity of all-ceramic posterior inlay-retained fixed dental prostheses. Eur. J. Oral Sci..

[44] Sarafidou K., Stiesch M., Dittmer M.P., Jörn D., Borchers L., Kohorst P. (2012). Load-bearing capacity of artificially aged zirconia fixed dental prostheses with heterogeneous abutment supports. Clin. Oral Investig..

[45] Sasse M., Krummel A., Klosa K., Kern M. (2015). Influence of restoration thickness and dental bonding surface on the fracture resistance of full-coverage occlusal veneers made from lithium disilicate ceramic. Dent. Mater..

[46] Yeslam H.E., Aljadaani A.K., Almalky A.M., Zahran M.M., Hasanain F.A. (2023). Effect of luting agent on the load-bearing capacity of milled hybrid ceramic single-tooth restoration. Ann. Dent. Spec..

[47] Zimmermann M., Ender A., Attin T., Mehl A. (2020). Fracture load of three-unit full-contour fixed dental prostheses fabricated with subtractive and additive CAD/CAM technology. Clin. Oral Investig..

[48] Alqarni H., Alfaifi M.A., Altoman M.S., AlHelal A.A., Magdy Ahmed W., Ahmed Azhari A., Kattadiyil M.T. (2024). A novel digital workflow for fabricating artificial periodontal ligament using three-dimensional printing flexible resin: A dental technique. Saudi Dent. J..

[49] Boeckler A.F., Morton D., Kraemer S., Geiss-Gerstdorfer J., Setz J.M. (2008). Marginal accuracy of combined tooth-implant-supported fixed dental prostheses after in vitro stress simulation. Clin. Oral Implant. Res..

[50] Bruschi-Alonso R.C., Alonso R.C., Correr G.M., Alves M.C., Lewgoy H.R., Sinhoreti M.A., Puppin-Rontani R.M., Correr-Sobrinho L. (2010). Reattachment of anterior fractured teeth: Effect of materials and techniques on impact strength. Dent. Traumatol..

[51] Clavijo V.G., Reis J.M., Kabbach W., Silva A.L., Oliveira Junior O.B., Andrade M.F. (2009). Fracture strength of flared bovine roots restored with different intraradicular posts. J. Appl. Oral Sci..

[52] Dulaimi S.F., Wali Al-Hashimi M.K. (2005). A comparison of spreader penetration depth and load required during lateral condensation in teeth prepared using various root canal preparation techniques. Int. Endod. J..

[53] Oliveira L., Melo C., Cavalcanti K., Soares P., Cardenas A., Soares C.J. (2022). Effects of adjacent tooth type and occlusal fatigue on proximal contact force of posterior bulk fill and incremental resin composite restoration. Oper. Dent..

[54] Piskin B., Aydin B., Sarikanat M. (2008). The effect of spreader size on fracture resistance of maxillary incisor roots. Int. Endod. J..

[55] Preis V., Behr M., Hahnel S., Rosentritt M. (2015). Influence of cementation on in vitro performance, marginal adaptation and fracture resistance of CAD/CAM-fabricated ZLS molar crowns. Dent. Mater..

[56] Soares P.B., Fernandes Neto A.J., Magalhaes D., Versluis A., Soares C.J. (2011). Effect of bone loss simulation and periodontal splinting on bone strain: Periodontal splints and bone strain. Arch. Oral Biol..

[57] Souza E.M., Calixto A.M., Lima C.N., Pappen F.G., De-Deus G. (2014). Similar influence of stabilized alkaline and neutral sodium hypochlorite solutions on the fracture resistance of root canal-treated bovine teeth. J. Endod..

[58] Tanomaru-Filho M., Sivieri-Araujo G., Guerreiro-Tanomaru J.M., Bortoluzzi E.A., Jorge E.G., Abi-Rached F.O., Reis J.M. (2014). Resistance of teeth with simulated incomplete rhizogenesis with intraradicular post or root canal filling. J. Contemp. Dent. Pract..

[59] Bömicke W., Waldecker M., Krisam J., Rammelsberg P., Rues S. (2018). In vitro comparison of the load-bearing capacity of ceramic and metal-ceramic resin-bonded fixed dental prostheses in the posterior region. J. Prosthet. Dent..

[60] Weigl P., Sander A., Wu Y., Felber R., Lauer H.-C., Rosentritt M. (2018). In-vitro performance and fracture strength of thin monolithic zirconia crowns. J. Adv. Prosthodont..

[61] Berthold C., Auer F.J., Potapov S., Petschelt A. (2011). In vitro splint rigidity evaluation—Comparison of a dynamic and a static measuring method. Dent. Traumatol..

[62] Berthold C., Auer F.J., Potapov S., Petschelt A. (2011). Influence of wire extension and type on splint rigidity—Evaluation by a dynamic and a static measuring method. Dent. Traumatol..

[63] Re D., De Angelis F., Augusti G., Augusti D., Caputi S., D’Amario M., D’Arcangelo C. (2015). Mechanical properties of elastomeric impression materials: An in vitro comparison. Int. J. Dent..

[64] Natali A., Pavan P., Carniel E., Dorow C. (2004). Viscoelastic response of the periodontal ligament: An experimental-numerical analysis. Connect. Tissue Res..

[65] Rees J.S. (2001). An investigation into the importance of the periodontal ligament and alveolar bone as supporting structures in finite element studies. J. Oral Rehabil..

[66] Qian L., Todo M., Morita Y., Matsushita Y., Koyano K. (2009). Deformation analysis of the periodontium considering the viscoelasticity of the periodontal ligament. Dent. Mater..

[67] Berthold C., Holst S., Schmitt J., Goellner M., Petschelt A. (2010). An evaluation of the Periotest method as a tool for monitoring tooth mobility in dental traumatology. Dent. Traumatol..

[68] Tricco A.C., Lillie E., Zarin W., O’Brien K.K., Colquhoun H., Levac D., Moher D., Peters M.D.J., Horsley T., Weeks L. (2018). PRISMA Extension for Scoping Reviews (PRISMA-ScR): Checklist and Explanation. Ann. Intern. Med..

[69] Zhang Z., Thompson M., Field C., Li W., Li Q., Swain M.V. (2016). Fracture behavior of inlay and onlay fixed partial dentures—An in-vitro experimental and XFEM modeling study. J. Mech. Behav. Biomed. Mater..

