# Supplementary material for: Simulation of the Periodontal Ligament in Dental Materials Research: A CAD/CAM-Based Method for PDL Modeling
Source: J Funct Biomater. 2025 Nov 24;16(12):429. doi: 10.3390/jfb16120429 (PMC12733424; doi:10.3390/jfb16120429)
Supplement: Supplementary file 1 [file jfb-16-00429-s001.zip › Supplementary_Table_S1_PDL_Checklist.pdf]

Supplementary Table S1. Minimum Reporting Checklist for Studies Including Periodontal Ligament (PDL) Simulation

Category	Recommended Reporting Item	Example / Note
PDL fabrication method	Describe the method used to create the PDL space and apply the analogue.	e.g., lost-wax technique, foil spacer, direct root coating, or CAD/CAM-based digital offset.
PDL analogue	Specify the material type, brand, and viscosity.	e.g., polyvinyl siloxane, putty (brand).
PDL layer thickness	Report the target value, tolerance, and verification method.	e.g., 0.70 ± 0.1 mm; verified using microcomputed tomography (µCT).
Functional validation	Specify the validation method and physiological target range.	e.g., Periotest device (target range 1–3 PTV), or Universal testing machine (deflection under 50 N axial load; target mobility range 50-80 µm)
Positioning and alignment	Describe the root-axis alignment procedure and use of positioning devices or jigs.	e.g., 3D-printed positioner or acrylic jig maintaining perpendicular root alignment
Tooth and substrate	Identify the type of tooth, number of roots and substrate material.	e.g., human maxillary molar, or composite resin (brand) incisor replica,
Socket material	Provide the composition, manufacturer, and method of cavity preparation.	e.g., polymethyl methacrylate (PMMA) block (brand), CAD/CAM-milled cavity generated by digital subtraction of the tooth model.
Cementation and restoration	Report the luting system, restorative material, and surface treatment.	e.g., lithium disilicate (brand) crown cemented with resin cement (brand) after hydrofluoric acid etching and silane (brand) application.
Aging and loading protocol	Describe thermocycling and mechanical fatigue parameters.	e.g., 500,000 cycles, 49 N load, 5–55 °C temperature range, 30 s dwell time.
Outcome measures	Define evaluated parameters and measurement techniques.	e.g., fracture resistance (N); universal testing machine
Uncertainty assessment	Identify potential error sources and report variability metrics.	e.g., seating angle deviation or material property inconsistency; report SD, 95% CI, or coefficient of variation (CV).
